# Cholecystitis Without Gallstones

**DOI:** 10.1155/1990/89848

**Published:** 1990

**Authors:** J-Y. Kang, R. C. N. Williamson

**Affiliations:** 1 Department of Surgery Royal Postgraduate Medical School Hammersmith Hospital London United Kingdom; 2 Department of Surgery Royal Postgraduate Medical School Hammersmith Hospital Du Cane Road London W12 0NN. United Kingdom; 3 Division of Gastroenterology Department of Medicine National University Hospital Singapore


*HPB Surgery* 1990, Vol. 2 pp. 83–103
Reprints available directly from the publisher
Photocopying permitted by license only

© 1990 Harwood Academic Publishers GmbH
Printed in the United Kingdom

### REVIEW ARTICLE
### CHOLECYSTITIS WITHOUT GALLSTONES

J-Y. KANG* AND R.C.N. WILLIAMSON

*Department of Surgery, Royal Postgraduate Medical School and Hammersmith Hospital, London, U.K.*

*(Received 29 May 1989)*

### INTRODUCTION

Some degree of cholecystitis is inevitably found in gallbladders that contain calculi. Yet both acute and chronic inflammation of the gallbladder can arise in the absence of stones. Acute acalculous choleycstitis is a dangerous disease that is sometimes encountered among seriously ill patients who are kept alive in a surgical intensive therapy unit. Chronic acalculous cholecystitis is a rather shadowy entity, but it certainly accounts for some patients with "typical" biliary pain in whom investigations are repeatedly negative for gallstones. Identification of such patients is difficult but worthwhile, because cholecystectomy is as likely to be curative as it is in calculous disease. Occasionallly there is a clear-cut cause for acalculous inflammation of the gallbladder, such as a specific bacteraemia or mechanical obstruction of the cystic duct. This review will consider the acute and chronic types of acalculous cholecystitis plus these secondary forms of the disease, terminating with a brief discussion of cholecystitis without gallstones in paediatric practice.

### ACUTE ACALCULOUS CHOLEYCYSTITIS

### *Epidemiology*

Approximately 10 per cent of all cases of acute cholecystitis develop in the absence of gallstones[1], precise figures ranging from 6–17 per cent in different series[2–5]. Acute acalculous cholecystitis (AAC) particularly affects patients who have undergone recent trauma or major surgical operations: in 3 large series between 12–49 per cent of cases of AAC belonged to one or other of these two categories[2, 6,7]. Amongst the remainder, underlying conditions of possible aetiological significance include cardiovascular disease, liver disease, systemic infections, diabetes mellitus, lupus erythematosus and polyarteritis nodosa[2,7]. In Lygidakis' experience of 80 patients, three other causative factors loomed large in AAC[7]. Papillitis was present in 25 per cent of patients, as demonstrated by histological examination and manometry, 6 per cent had had previous truncal vagotomy (presumably causing biliary stasis) and in 18 per

Correspondence to: Professor R.C.N. Williamson, Department of Surgery, Royal Postgraduate Medical School, Hammersmith Hospital, Du Cane Road, London W12 0NN., UK.
* Present address: Division of Gastroenterology, Department of Medicine, National University Hospital, Singapore.

cent the cystic duct was described as long and tortuous (difficult criteria to define) and thus a potential cause of outflow obstruction from the gallbladder. According to Glenn[2], however, up to half the cases of AAC are idiopathic, i.e. they develop spontaneously in the absence of concomitant disease.

Increasingly AAC is recognised as a disease of surgical patients in hospital. Among postoperative patients or those with recent trauma, between 33 and 100 per cent of attacks of acute cholecystitis are acalculous[8–16] compared to 10 per cent of attacks complicating medical illnesses or developing overall[12]. The actual prevalence of post-traumatic or postoperative AAC depends upon the clinical setting. It affects 1 in 25,000 patients if all surgical procedures are considered[8] or 0.1–1.6 per cent of those requiring intensive care following major operations in general or cardiac surgery[17–19]. AAC developed in 0.3% of patients undergoing elective repair of an aortic aneurysm compared to 13.6% of patients requiring emergency operation for ruptured aneurysm[20]. Among war casualties AAC occurs in 0.5 per cent of cases[9], while in victims of major burn injuries the proportion rises to 3.5 per cent[21].

Gallstones and thus calculous cholecystitis are commoner in women, but there is a slight male preponderance (up to 1.5:1) for AAC overall[2,6]. The male/female ratio rises to 7:1 for postoperative cases and reaches 20:1 for post-traumatic patients[22]. By contrast, the sex ratios for acute calculous cholecystitis approach unity in those with recent operations or trauma[22]. This discrepancy could indicate that acute calculous cholecystitis in these settings is coincidental and unrelated to the recent injury[13]. Alternatively, factors that promote the development of AAC also provoke acute cholecystitis in gallbladders with pre-existing stones, the calculi being incidental[23]. Patients with post-traumatic AAC tend to be younger than patients with postoperative AAC[22], probably because trauma victims tend to be young men[13].

AAC can occur from a few days to several months after the operation or injury but usually within 2–4 weeks[16]. Postoperative AAC is especially likely after gastrointestinal surgery[16], in those with surgical complications24 and in those with parenteral nutrition[3].

There is evidence that AAC is becoming more frequent in recent years. In 1982 Glenn and Becker reported an increase over a 30-year period in both the number of cases of AAC as well as the proportion of acalculous cases amongst patients with acute cholecystitis[23]. Petersen and Sheldon also found an increase in the frequency of AAC over a 6-year period in their institution[3]; this increase paralleled the growing use of parenteral nutrition. Likewise Gutman reported an increase in the frequency of acalculous cholecystitis, both acute and chronic, over a fifteen-year period[25].

*Aetiology*

Relevant aetiological factors in AAC are summarised in Table 1. Many of these factors are interrelated, and they may summate in patients who are critically ill. Animal experiments indicate that cystic duct obstruction, concentrated bile and impaired blood supply are each important in the development of acute cholecystitis[26]. In the rabbit, bile acids themselves can damage the gallbladder mucosa, this effect being prevented by lecithin[27]. When compared to normal, patients with cholecystitis have more deoxycholic acid and less lecithin in the bile[27]. Bacterial invasion is generally superimposed on injured tissue[28]. In the absence of calculi, bile stasis is believed to cause functional obstruction of the cystic duct. With reduced bile flow, the bile becomes increasingly concentrated in the gallbladder and bile viscosity rises. Viscid stagnant gallbladder contents may not readily pass through the cystic duct. In the postoperative or traumatised patient, fasting would be an important cause of bile stasis since it removes the physiological stimulus to gallbladder contraction. Gallbladder stasis is greater after intra-abdominal operations than extra-abdominal operations[29]. This finding is probably related to delayed resumption of oral intake after laparotomy and fits in well with the observation that AAC tends to complicate gastrointestinal operations[10,16,22]. Truncal vagotomy impairs biliary motility in some but not all patients[30,31] and has been incriminated as another aetiological factor in AAC[7,15].

**Table 1 – Possible aetiological factors for acute acalculous cholecystitis**

**Bile stasis**
- postoperative or post-trauma state
- fasting and dehydration
- sustained fever
- prolonged opiate therapy
- mechanical ventilation
- parenteral nutrition
- truncal vagotomy
- papillitis
- resumption of feeding (in the face of functional obstruction)

**Alteration in bile composition**
- multiple blood transfusions
- resorption of sequestered blood and haematomas

**Ischaemia**
- arteriosclerosis
- hypotensive episodes
- vasomotor drugs
- sympathetic activity

**Infection**
- specific (see Table 4)
- non-specific

**Mechanical obstruction**
(see Table 4)

**Others**
- activation of factor XII-dependent pathway
- increase in prostaglandin E levels in gallbladder mucosa

Several other factors contribute to bile stasis in surgical patients. Fever and dehydration will increase bile viscosity. Opiate drugs have a spasmogenic effect on the sphincter of Oddi and will thus further promote biliary stasis[32–34]. Assisted ventilation with positive end-expiratory pressure will also reduce bile flow into the duodenum[35] and may therefore help to cause AAC. Parenteral nutrition has recently been found to be a risk factor for the development of both calculous and acalculous cholecystitis[3,19,36]. Absence of oral intake and thus impaired Cholecystokinin (CCK) secretion is probably the dominant factor leading to bile stasis in this situation. Glenn and Wantz had previously described a temporal relationship between resumption of feeding, when the gallbladder presumably tries to evacuate its contents, and the onset of AAC[37]. Although Levin reported similar findings[38], subsequent authors have been unable to confirm this relationship[11,12,16,39]. Patients in an intensive therapy unit often receive both ventilation, parenteral nutrition and regular opiates and are thus good candidates for AAC.

Gallbladder stasis in postoperative or otherwise acutely ill patients can theoretically be prevented or treated by resumption of food intake or by administration of a cholecystagogue. In the dog model, daily infusions of cholecystokinin octapeptide prevented the gallbladder stasis resulting from total parenteral nutrition[40]. In a group of patients who had undergone oesophagectomy, enteral feeding via an enterostomy as early as the fifth postoperative day prevented or reduced biliary stasis and development of debris in the gallbladder[41].

In some series of AAC many patients had received large-volume blood tranfusions[9,34]. It was suggested that the increased bile pigment load secondary to transfusion may injure the gallbladder. A similar effect may occur in the resorption of sequestered blood from fracture sites and haematomas in trauma patients. However, Long found no differences in the amount of blood transfused in patients with AAC compared to control subjects[19].

Once biliary stasis and functional obstruction have developed, either progressive distension of the gallbladder or forceful contraction could start a vicious cycle whereby venous and lymphatic channels in the wall are compressed. Even the arterial supply might be compromised by mural oedema. Ischaemia would increase the susceptibility of the gallbladder to noxious agents such as toxins, enzymes, chemical agents and bacteria. Ischaemia could be a major risk factor for AAC in elderly patients with arteriosclerosis and in critically ill patients with episodes of hypotension, especially if vasoactive drugs are administered. Sympathetic overactivity during shock may further constrict gallbladder blood vessels. Sympathectomy protects against experimental acute cholecystitis in dogs[42], while hypotensive shock can cause focal necrosis of the gallbladder wall[43].

As in acute calculous cholecystitis[44], infection is probably not a direct cause of AAC but rather a secondary phenomenon[14,45]. Certainly, a proportion of bile cultures in every series turns out to be sterile. However, the presence of open wounds is said to predispose to AAC[19]. In two other series, 8 of 9 patients with AAC had had bacteraemia in the preceeding week,[39] and 6 of 9 patients with positive bile cultures had previous wound infections with the same organism[9]. Therefore infection could have a primary role in the causation of AAC in some cases.

Becker suggested the involvement of Factor XII-dependent pathways in the pathogenesis of AAC[46]. These pathways may be activated by the transfusion of blood products or by gram-negative bacterial toxins. He demonstrated intense injury to blood vessels in the muscularis and serosa of dog gallbladders by the systemic injection of Factor XII activators. Other abdominal viscera were affected to a lesser degree or not at all.

Concentrations of prostaglandin E but not prostaglandin F in the gallbladder were noted to be higher in AAC when compared to controls or patients with calculous disease[47,48]. These levels correlated with the severity of histological inflammation, so prostaglandins could mediate the inflammatory response[49].

*Clinical Features*

The symptoms and signs of AAC are essentially the same as those of acute calculous cholecystitis, except perhaps that constitutional upset tends to be greater[6,16,24,34]. However, the typical features may be overshadowed by the concomitant illness or be masked in injured postoperative patients who are heavily sedated and mechanically ventilated. Abdominal pain, commonly in the right upper quadrant, fever and vomiting are the symptoms most frequently reported. Physical signs include abdominal tenderness, distension, loss of bowel sounds and occasionally jaundice. It is important to remember that AAC may present purely as a persistent unexplained fever or as a sudden deterioration in general health. Leucocytosis, hyperamylasaemia and abnormal liver function tests may occur, but they are neither constant nor specific features[6,34]. A high index of clinical suspicion is therefore needed if this potentially lethal condition is to be diagnosed before the onset of complications.

*Diagnostic Tests*

The plain abdominal film is unhelpful in the diagnosis of AAC, while oral cholecystography is inappropriate in acutely ill patients who may be nauseated and vomiting. Ultrasonography is non-invasive and safe. It can also be performed at the bedside using a portable machine. Thickening of the gallbladder wall, gallbladder distension, subserosal oedema, pericholecystic abscess, biliary sludge and wall fragmentation are all helpful clues[50,51]. Individual signs vary in sensitivity and specificity. Wall thickening, for example, occurs in 81–90 per cent of cases of AAC[50,51] but is also found in non-biliary conditions including hepatitis, hypoproteinaemia, heart failure, renal disease and myeloma[52]. Experienced operators may be able to differentiate between the mural oedema of a hypoproteinaemic state and the inflammatory thickening of cholecystitis[53]. Serial scans allow luminal distension and wall thickening to be closely monitored.

Cholescintigraphy involves the intravenous injection of a technetium- labelled derivative of iminoacetic acid. Non-visualisation of the gallbladder despite good hepatic uptake and isotope entry into the intestine indicates gallbladder disease. Free intraperitoneal spill of isotope suggests perforation[5,54,55]. Impaired hepatic function may vitiate the test.

The sensitivity and specificity of both ultrasonography and scintigraphy have been assessed by several groups. Mirvis reported a sensitivity of 92 per cent and a specificity of 96 per cent in the ultrasonic diagnosis of AAC[56], but other workers using different criteria reported lower sensitivities varying from 36–89 per cent[6,57–60]. By contrast, the sensitivity of scintigraphy in the diagnosis of AAC is consistently high, figures of 83–100 per cent being reported[57–60]. Yet, specificity seems to be a problem, with up to 54 per cent of abnormal scans being false positive[56]. The specificity of scintigraphy in the diagnosis of acute calculous cholecystitis is much better (81–96%)[61,62].

In a small series of 15 patients, computerised axial tomography was found to be 100 per cent sensitive and specific in the diagnosis of AAC[56], but these results obviously need confirmation. Other techniques of potential value in the diagnosis of AAC include percutaneous aspiration of bile from the gallbladder and indium-labelled leucocyte scanning[56,63,64].

Preoperative diagnosis is certainly more difficult in AAC than it is in calculous disease, and this difficulty must contribute to the high prevalence of complications. In one large series, for example, 48 per cent of patients showed areas of gangrene while perforation occurred in 8 per cent and empyema in 6 per cent[24]. In another large series, 50 per cent of patients with AAC had gangrenous gallbladders[6], and 40 per cent of 35 gallbladders perforating during the course of acute cholecystitis had inflammation in the absence of stones[65]. Johnson recently reported that perforation occurred five times more frequently if operation was delayed until 48 hours after the onset of symptoms than if no delay in surgical treatment occurred[66].

### *Treatment*

Although patients with AAC are sometimes given a trial of medical treatment, the attendant toxicity and the risk of complications mean that early laparotomy is essential unless there is rapid clinical improvement. Because gangrene is so common, except in children, cholecystectomy is usually a better option than cholecystostomy. Subtotal cholecystectomy, leaving the adherent posterior wall of the gallbladder, is a reasonable alternative in the presence of gross inflammation[67]. Nevertheless, some patients have been successfully treated by conservative measures alone[12,68]. Ultrasound-guided percutaneous drainage of the gallbladder can be performed under local anasthesia as a bedside procedure. In a small experience all 6 patients thus treated improved without complications, so this technique deserves further evaluation[63]. Some patients may recover after cholecystostomy without the need for subsequent cholecystectomy[19,21]. In most series, cholecystectomy ensures a better survival rate than cholecystostomy[2,19,34], probably because the lesser procedure is chosen for the gravely ill.

The overall mortality rate of AAC is 15 per cent (91 of 594 cases) in 33 series, rising to 27 per cent (66 of 245 cases) when only post-traumatic or postoperative cases are considered[2,3,6–9,12–15,17–24,34,37–39,43,55,63,66,68–74]. Postoperatively, more than twice as many patients with AAC die than those with calculous acute cholecystitis[8].

### CHRONIC ACALCULOUS CHOLECYSTITIS

### *Definition and Clinical Features*

Chronic acalculous cholecystitis (CAC) is a poorly defined clinical entity. Strictly speaking, the term should only be applied when there is chronic inflammation of the gallbladder on histological examination. But sometimes the term CAC is loosely taken to include other ill-defined acalculous conditions of the gallbladder, such as motility disorders, cholesterosis or adenomyosis, in which inflammatory changes are trivial or absent[44,75]. Unfortunately chronic biliary tract symptoms correlate poorly with the macroscopic and microscopic appearances of the gallbladder.

Although biliary colic is a symptom that clearly implicates the gallbladder, the same may not be true for dyspepsia. Price found that dyspepsia was just as common in women with normal cholecystograms as it was in those with gallstones[76]. By contrast, Rhind described good symptomatic relief of dyspepsia following cholecystectomy[77]. Although most authors suggest that CAC can cause dyspeptic symptoms[78–80], patients with biliary colic are more likely than others to benefit from cholecystectomy[81].

From historical reports it appears that cholecystitis is frequently asymptomatic. In a series of macroscopically normal gallbladders resected in patients with non-biliary disorders, many neutrophils were observed histologically in 48 per cent[82]. Likewise, in an autopsy series in which only 8 per cent of deaths were attributable to gallbladder disease, 62 per cent of gallbladders were macroscopically diseased while a further 13 per cent were macroscopically normal but histologically inflamed[83]. Conversely a healthy gallbladder can theoretically cause pain if there is obstruction to bile flow. Grossly thick-walled gallbladders may turn out to be histologically normal[84], though criteria for the histological diagnosis of chronic cholecystitis seem to vary between observers[78]. The pathological appearances of cholecystectomy specimens cannot be distinguished between patients with and without persistent symptoms[84].

The symptoms of CAC overlap with those of cholelithiasis[84]; physical examination and laboratory tests are similarly unhelpful in differentiating between the two conditions. The oral cholecystogram in CAC may be normal or show impaired opacification or filling defects[84]. The findings on ultrasonography and scintigraphy are equally non-specific. Ultrasound examination, for example, is only 29 to 62 per cent sensitive for CAC[58,59,79,85], while scintigraphy can be normal in as many as 60 per cent of cases[79,86].

Irrespective of whether clinical or histological criteria are used (and in contrast to AAC), most series of CAC show a marked female preponderance with a sex ratio varying from 2.5:1 to 10:1[79,80,87].

*Provocative Testing and Cholecystectomy*

Since preoperative diagnosis of CAC is difficult and the symptomatic response to cholecystectomy unpredictable, provocative tests have been devised to try and identify patients who will benefit from operation. A provocative agent is administered, and one or more of the following are taken to imply gallbladder abnormality:
1) reproduction of abdominal pain, 2) impaired contraction of the gallbladder demonstrable by oral cholecystography, scintigraphy or ultrasonography, or 3) absence of gallbladder bile on duodenal aspiration or demonstration of pus cells, cholesterol or bilirubin crystals in the bile.

The most commonly used provocative agent is cholecystokinin (CCK). By stimulating contraction of the gallbladder and relaxation of the sphincter of Oddi, this hormone produces gallbladder emptying in healthy individuals[88,89]. Ivy first noted that CCK administration could produce biliary colic in some patients and might therefore facilitate the diagnosis of acalculous biliary disease[32]. The pain typically occurred during emptying of the gallbladder and presumably arose from contraction of an inflamed viscus. Subsequently it was suggested that CCK also induced biliary pain in some patients with normal gallbladders in whom bile flow was impeded by pathological narrowing of the cystic duct, the so-called cystic duct syndrome[90–93].

Some authorities report that patients with acalculous biliary disease have reduced gallbladder emptying in response to CCK when compared to healthy subjects[94–96], but others do not[97,98]. This finding has been taken to imply gallbladder disease even if abdominal pain is not reproduced[91,99,100]. It has been suggested that patients with a decreased gallbladder response to CCK are more likely to develop gallstones at a later stage[101]. An alternative mechanism for biliary pain after CCK administration is through spasm of the sphincter of Oddi[102,103]. Yet even in healthy subjects CCK can produce hypertonicity of the gallbladder infundibulum, with consequent delay in gallbladder evacuation and associated pain, especially if the hormone is administered too rapidly[89,104]. CCK has extra-biliary effects too, increasing motility in both small and large intestine[105,106].

A typical provocation test involves the intravenous administration of 1 Ivy dog unit/Kg body weight of CCK over a period of 30 seconds to three minutes while the occurrence of abdominal pain is noted. Gallbladder emptying can be assessed by means of cholecystography[107,108], ultrasonography[109] or scintigraphy[92,110]. The test can be refined by using a duodenal tube to aspirate bile for microscopic examination[99,111]

Preparations of CCK produced by KabiVitrum and Boots have been used by most investigators. More recently, sincalide (an octapeptide of CCK) or ceruletide (a structurally similar decapeptide) have also been used; each agent produces gallbladder contraction[104,112–116]. Sincalide is more convenient and more effective by the intramuscular route than the intravenous route[112,117]. A fatty meal has also been used to produce CCK release and hence gallbladder contraction[118,119]. Hopman and co-workers found fatty meals to be of equivalent potency to exogenous CCK in causing gallbladder contraction[120], but Park found fatty meals to be less effective[121]. Certainly, the rate of gastric emptying is variable and different fatty meals have different potencies[122].

It is generally accepted that these provocative tests are useful in selecting patients with suspected biliary pain but without demonstrable calculi for cholecystectomy. In most series 80 per cent or more of patients with positive CKK provocation tests have been improved by cholecystectomy (Table 2). This result compares well with that of cholecystectomy for gallstone disease. At laparotomy the gallbladder could be either normal or inflamed on gross examination; the cystic duct was sometimes found to be narrow or tortuous. Microscopic examination of the gallbladder commonly showed chronic cholecystitis, but some patients have had normal gallbladders and a few others have had cholecystoses or calculous disease. Surprisingly the symptomatic response is independent of the appearance of the gallbladder, whether operative or histological[97,123–127]

There are several drawbacks to the use of the CCK provocation test. Several authors have questioned its specificity, since CCK causes pain in some healthy individuals possibly as a result of infundibular spasm[97,98]. In particular, Dunn and colleagues provoked biliary-type pain in 27 per cent of healthy controls[123]. Both Dunn and Nathan gave CCK over 15–45 seconds[98,123], and such rapid injection may lead to spasm of the gallbladder neck in normal individuals[94]. Further, CCK is known to increase intestinal motility and cause abdominal pain in patients with irritable bowel syndrome[128]. Since irritable bowel syndrome is an important differential diagnosis to CAC in patients with otherwise unexplained abdominal pain, biliary pain might conceivably be confused with intestinal pain. Lastly, a positive CCK test is not always reproducible on repeat testing[97], and radiological interpretation of impaired gallbladder emptying is subject to considerable observer bias[123].

Proper evaluation of the CCK test would involve comparison of operative and conservative treatment in both positive and negative patients. Most authors, but not all, report that positive responders to the CCK test fare better with operation than prolonged conservative therapy (Table 2)[91–95, 97,99,100,111,123,125–127,129–139]. Among the fewer negative responders submitted to cholecystectomy, overall results have been slightly inferior to those of cholecystectomy for positive responders, yet symptomatic benefit has still been reported in 80 per cent or more of such patients (Table 3)[97,111,123,125,126,131,134,135,137–142]. Moreover, even negative responders to CCK seem to fare better after cholecystectomy than on conservative management alone. It may be that cholecystectomy has a non- specific effect on abdominal pain irrespective of the results of the CCK test. We therefore agree with Sunderland and Carter[143] and Berk[144] that routine use of the CCK test for selection of patients with suspected biliary pain for cholecystectomy is of unproven value, although no better test is currently available.

*Non-surgical Treatment of CAC*

Hypertonicity of the gallbladder neck induced by CCK can be ameliorated by the use of amyl nitrite or glyceryl trinitrate[32,89]. These antispasmodics, together with a low fat diet, have been used in patients with CAC with generally unsatisfactory results[93,94,127,129]. It has recently been suggested that precipitation of cholesterol crystals in CAC may represent an early stage of calculous disease[130,145]. If this hypothesis were to be confirmed, dissolution therapy could in theory be an attractive proposition.

### SECONDARY ACALCULOUS CHOLECYSTITIS

While most cases of acalculous cholecystitis are idiopathic, a small proportion can be clearly attributed to an underlying illness or agent (Table 4).

*Specific infections and infestations*

These are important causes of acalculous cholecystitis in certain clinical settings. In one report from India ascariasis was the aetiological agent in 40 of 87 patients with biliary tract disease[146]. Most of these patients presented with recurrent pyogenic cholangitis, but 9 had acalculous cholecystitis; calculous disease was only seen in 38 patients. Other uncommon infectious causes of acalculous cholecystitis in previously healthy individuals include Salmonella typhi[147,148], Staphylococcus aureus[149], haemolytic streptococcus[148], Leptospira icterohaemorrhagiae[150] and Schistosoma mansoni[151]. Tuberculous cholecystitis can also result from cystic duct obstruction by necrotic material[152]. Ultrasonic features of acalculous cholecystitis were reported in a patient with hepatitis A who recovered uneventfully[153]. Emphysematous cholecystitis, in which the infecting organisms (e.g. clostridia, coliforms) produce gas, is occasionally seen in the absence of gallstones[154,155].

The biliary tract can be involved by opportunistic infections in the acquired immunodeficiency syndrome. Presentation can be with abdominal pain, fever, jaundice, or merely with abnormal liver function tests. Both Candida albicans and cytomegalovirus have been reported to cause acalculous cholecystitis in this setting[156–158]. Acalculous cholangitis demonstrable radiologically has also been attributed to superinfection with Cryptosporidium[159,160].

*Mechanical Obstruction*

Rarely, acalculous cholecystitis can result from cystic duct obstruction by tumour. Primary carcinoma of the gallbladder[161] or cystic duct[162], as well as secondary breast carcinoma[163], can each present as AAC. Obstruction by secondary melanoma[164] and Hodgkin's disease[165] can cause CAC. Another patient with acquired immunodeficiency syndrome developed fever and jaundice owing to obstruction of the cystic duct by Kaposi's sarcoma[157].

Torsion of the gallbladder is a rare cause of necrotizing AAC. It typically occurs in elderly women, but any age group can be affected[166,167]. The age at presentation may be related to the underlying anatomy. Thus two anomalies that

**Table 2 – Response to therapy in patients with positive cholecystokinin provocation tests**

| Author | CHOLECYSTECTOMY | | | | CONSERVATIVE TREATMENT | |
|---|---|---|---|---|---|---|
| | *No.* | *% abnormal gallbladder or cystic duct* | *No. followed up* | *% symptomatic improvement* | *No.* | *% symptomatic improvement* |
| Burnstein* 1982[131] | 24 | 92 | 21 | 100 | 4 | 0 |
| Byrne* 1985[125] | | | 21 | 43 | 11 | 45 |
| Conte* 1971[91] | 7 | 100 | | | | |
| Davis* 1982[97] | | | 7 | 86 | | |
| Dunn 1974[123] | | | 19 | 89 | | |
| Einarsson 1986[132] | 15 | 67** | 15 | 93** | | |
| Fink-Bennet* 1985[92] | 14 | 100 | 14 | 100 | | |
| Foss* 1977[111] | 26 | 100** | | | | |
| Freeman* 1975[99] | 22 | 64 | 22 | 100 | | |
| Goldberg 1976[133] | | | 15 | 93 | | |
| Goldstein 1974[94] | 23 | 78 | 17 | 65 | | |
| Griffen* 1980[100] | 16 | 100 | 16 | 94 | | |
| McFarland* 1969[93] | 9 | 100 | 9 | 100 | | |
| Moskovitz* 1986[134] | | | 26 | 81** | 10 | 40 |
| Nathan* 1970[135] | 79 | 97** | 70 | 96** | | |
| Neschis 1978[129] | 19 | 74** | 18 | 94 | | |
| Newman* 1983[95] | 7 | 86 | 7 | 100 | | |
| Nora* 1974[136] | 10 | 100 | 10 | 100 | | |
| Nora* 1984[130] | 30 | 100** | 30 | 100 | | |
| Reid 1975[126] | 4 | 50 | 4 | 75 | 1 | 0 |
| Rhodes 1988[137] | 80 | 71 | 81 | 91 | | |
| Sunderland 1987[138] | | | 5 | 80 | | |
| Sykes 1982[139] | 15 | 100** | 15 | 93** | | |
| Valberg 1971[127] | 12 | 75 | 12 | 100 | | |

* pain or abnormal gallbladder emptying or abnormal duodenal bile taken to be a positive test

** some patients with calculi included

**Table 3 – Response to therapy in patients in patients with negative cholecystokinin provocation tests**

| *Author* | CHOLECYSTECTOMY | | | | CONSERVATIVE TREATMENT | |
|---|---|---|---|---|---|---|
| | *No.* | *% abnormal gallbladder or cystic duct* | *No. followed up* | *% symptomatic improvement* | *No.* | *% symptomatic improvement* |
| Burnstein* 1982[131] | 9 | 33 | 9 | 89 | 22 | 23 |
| Byrne* 1985[125] | | | 3 | 33 | 13 | 62 |
| Davis* 1982[97] | | | 2 | 50 | | |
| Dunn 1974[123] | | | 9 | 67 | | |
| Foss* 1977[111] | 5 | 0 | | | | |
| Madsen 1981[140] | | | 5 | 100 | | |
| Moskovitz* 1986[134] | | | 10 | 50** | 27 | 48 |
| Nathan 1970 [135] | 8 | 75 | | | | |
| Reid 1975[126] | 4 | 100** | 4 | 75** | 3 | 33 |
| Rhodes 1988[137] | | | | | 59 | 15 |
| Sunderland 1987[138] | | | 18 | 83 | | |
| Sykes 1982[139] | | | | | 1 | 0 |
| Thornell 1985[141] | | | 6 | 83 | | |
| Windsor 1982[142] | | | | | 7 | 57 |

* pain or abnormal gallbladder emptying or abnormal duodenal bile taken to be a positive test

** some patients with calculi included permit gallbladder torsion have been described[168]. In the first variety, presumably acquired, the gallbladder is suspended by a mesentery that is postulated to elongate because of visceroptosis in elderly subjects. In the second type, presumably congenital, the gallbladder is free-floating on the cystic duct and artery with no attachment to the liver. Exceptionally, torsion can be confined only to the fundus[168] or affects only one half of a double gallbladder[169]. In another case a floating gallbladder became incarcerated within an epigastric hernia[170]. Sudden onset of upper abdominal pain and vomiting are the typical features of torsion, pain often radiating to the back. A rare variant is that of partial recurrent torsion[166]. Since torsion of the gallbladder presents like severe acute cholecystitis, precise preoperative diagnosis is almost never achieved. Sonographic and computed tomographic appearances are non-specific[171]. Gangrene and perforation are almost inevitable in torsion, so prompt operation is needed in any patient with presumed acute cholecystitis who fails to settle.

**Table 4 – Causes of secondary acalculous cholecystitis**

**1. Specific infections and infestations**
- bacterial e.g. typhoid, leptospira, streptococcus, staphylococcus
- viral eg. hepatitis A
- helminthic e.g. ascaris, schistosoma
- gas-forming e.g. clostridia, coliforms (in emphysematous cholecystitis)
- opportunistic e.g. crytosporidium, cytomegalovirus, candida (in AIDS)

**2. Systemic diseases**
- collagen diseases e.g. polyarteritis nodosa, systemic lupus erythematosus, scleroderma
- mucocutaneous lymph node syndrome
- ulcerative colitis/sclerosing cholangitis
- Sjögren's disease

**3. Obstructive**
- mechanical e.g. torsion, stent, cystic duct stenosis
- primary tumour e.g. cystic duct, gall bladder
- secondary tumour e.g. Hodgkin's disease, melanoma, breast carcinoma, Kaposi's sarcoma

**4. Toxic**
- chemotherapy
- ceftriaxone
- lipiodol

We have encountered one case of acalculous cholecystitis associated with an indwelling endoprosthesis in a 67-year-old man with chronic pancreatitis. Following endoscopic papillotomy a stent had successfully been inserted to relieve jaundice caused by distal bile-duct stricture. At operation 6 weeks later patchy necrosis of the gallbladder wall was observed.

### *Systemic Diseases*

Acalculous cholecystitis can be associated with several collagen diseases. Polyarteritis nodosa may rarely present with AAC[172], which can also complicate systemic lupus erythematosus[7,173]. Acute hydrops of the gallbladder occurs in both the mucocutaneous lymph node syndrome[174] and Sjögren's syndrome[175]. Abnormalities in gallbladder histology have been reported in patients with sclerosing cholangitis[176] and scleroderma[177].

*Chemical Causes*

Hepatic artery infusion chemotherapy used in recent years for the treatment of hepatic metastases can damage those parts of the biliary tract directly supplied by this artery; the common bile duct is spared[178]. Acute or chronic cholecystitis can present with upper abdominal pain exacerbated by drug infusions[179,180]. In some patients sclerosis and narrowing of the proximal bile ducts is demonstrable radiologically and can cause jaundice[178,181]. At operation the gallbladder is often shrunken and fibrotic, but it may be actively inflamed[178–181] and surrounding structures are often involved. Floxuridine (5 FUDR) is the agent most often implicated (perhaps related to frequency of use), but mitomycin C and cisplatin can also be injurious.

Biliary complications are uncommon with intermittent chemotherapy delivered via percutaneously inserted catheters[179]. However, intensive regimes using implanted intra-arterial pump systems are thought to cause some degree of biliary damage in most patients undergoing prolonged therapy, as evidenced by elevation of serum alkaline phosphatase and transaminase levels[181]. In one series, all 6 patients with intact gallbladders required re-operation for acute cholecystitis or jaundice within nine months of pump implantation[178]. Sometimes symptoms subside on cessation of chemotherapy, even with the gallbladder in situ[179]. Nevertheless, it is wise to perform prophylactic cholecystectomy at the time of laparotomy for hepatic artery cannulation[178,181].

The use of ceftriaxone, a third generation cephalosporin, has recently been associated with the development of gallbladder sediments in up to 40 per cent of patients receiving high-dose therapy[182]. Biliary symptoms may or may not be present. Both symptoms and ultrasonographic appearances are reversible on cessation of therapy. We have lately seen AAC in one patient after injection of lipiodol into the hepatic artery to demonstrate liver tumour.

### ACALCULOUS CHOLECYSTITIS IN INFANCY AND CHILDHOOD

Cholecystitis is much more commonly acalculous in young children than it is in adults. Thus 13 of 16 children (81%) with acute cholecystitis in one series had no gallstones[183]. Literature reviews suggest that approximately 40 per cent of cases of acute cholecystitis in the paediatric age group are acalculous[184,185]. By contrast, in another series gallstones were found in 87 per cent of patients, but these were mainly older children up to age 20[186]. Young children with acalculous cholecystitis are predominantly male[45].

Sixty per cent of children with AAC have had a preceding systemic illness such as leptospirosis, scarlet fever or non-specific diarrhoea[45,148]. Mucocutaneous lymph node syndrome is another recognised cause in this age group[174]. In other cases cholecystitis could be related to congenital malformation of the bile ducts[45,187]. As in adults, the aetiology is generally obscure. Bile stasis could follow dehydration or total parenteral nutrition. Mechanical blockage could result from congenital stenosis of the cystic duct or ductal occlusion by enlarged lymph nodes. The gallbladder can vary from being distended but non-inflamed (i.e.acute hydrops) to necrotizing cholecystitis[188,189], but gangrene and perforation are said to be uncommon[45]. Bile culture may either be sterile or yield one of a wide range of pathogens.

The clinical presentation of AAC in children is similar to the adult disease. Abdominal pain is present in almost all cases[45,183] and can be localised or diffuse. Vomiting is frequent, and physical signs include tenderness, fever, mass and jaundice. Preoperative diagnosis is not always possible, but abdominal ultrasound examination is of particular value in this age group[188,190,191].

Although acute hydrops of the gallbladder may resolve spontaneously, perforation and biliary peritonitis can occur[187], so decompression is recommended. Simple percutaneous aspiration of the gallbladder may be sufficient treatment. Percutaneous cholecystostomy is another recommended approach[45] and should be followed by secondary cholecystectomy if indicated by cholangiographic findings; primary cholecystectomy also has its advocates[45,190]. The mortality rate can reach 20 per cent in neonates[192], but two larger series totalling 23 (mainly older) children included no deaths[183,186].

### *References*

1. Williamson, R.C.N., (1988). Acalculous disease of the gallbladder. Gut **29**, 860–872.
2. Glenn, F. (1979). Acute acalculous cholecystitis. *Annals of Surgery* **189**, 458–465.
3. Petersen, S.R. and Sheldon, G.F. (1979). Acute acalculous cholecystitis: a complication of hyperalimentation. *American Journal of Surgery* **138**, 814–817.
4. Pickleman, J., Gonzalez, R.P. (1986). The improving results of cholecystectomy. *Archives of Surgery* **121**, 930–934.
5. Swayne, L.C. (1986). Acute acalculous cholecystitis: sensitivity in detection using technitium-99$^{m}$ Iminodiacetic acid cholescintigraphy. *Radiology* **160**, 33–38.
6. Fox, M.S., Wilk P.J., Weissmann, H.S., Freeman, L.M. and Gliedman, M.L. (1984). Acute acalculous cholecystitis. *Surgery Gynecology and Obstetrics* **159**, 13–16.
7. Lygidakis, N.J. (1981). Surgery for acalculous cholecystitis. *American Journal of Gastroenterology* **76**, 27–31.
8. Devine, R.M., Farnell, M.B. and Mucha, P.Jr. (1984). Acute cholecystitis as a complication in surgical patients. *Archives of Surgery* **119**, 1389–1393.
9. Lindberg, E.F., Grinnan, G.L.B. and Smith, L. (1970). Acalculous cholecystitis in Vietnam casualties. *Annals of Surgery* **171**, 152–157.
10. Thompson, J.W., Ferris, D.O. and Baggenstoss, A.H. (1962). Acute cholecystitis complicating operations for other diseases. *Annals of Surgery* **155**, 489–494.
11. Ottinger, L.W. (1976). Acute cholecystitis as a postoperative complication. *Annals of Surgery* **184**, 162–165.
12. Gately, J.F. and Thomas E.J. (1983). Acute cholecystitis occurring as a complication of other diseases. *Archives of Surgery* **118**, 1137–1141.
13. Fabian, T.C., Hickerson, W.L. and Mangiante, E.C. (1986) Post traumatic and postoperative acute cholecystitis. *American Surgeon* **52**, 188–192.
14. DuPriest, R.W., Khaneja, S.C. and Cowley, R.A. (1979). Acute cholecystitis complicating trauma. *Annals of Surgery* **189**, 84– 89.
15. Ziv, Y., Feigenberg, Z., Zer, M. and Dintsman, M. (1987) Acute cholecystitis complicating unrelated disease: etiological considerations. *American Journal of Gastroenterology* **82**, 1165–1168.
16. Kasahara, Y. Umemura, H., Kuyama, T. and Oku, H. (1978). Postoperative acute choleycstitis in Japan. *World Journal of Surgery* **2**, 661–666.
17. Savino, J.A., Scalea, T.M. and DelGuercio, L.R.M. (1985). Factors encouraging laparotomy in acalculous cholecystitis. *Critical Care Medicine* **13**, 377–380.
18. Welling, R.E., Rath R., Albers, J.E. and Glaser, R.S. (1986). Gastrointestinal complications after cardiac surgery. *Archives of Surgery* **121**, 1178–1180.
19. Long, T.N., Helmbach, D.M. and Carrico, C.J. (1978). Acalculous cholecystitis in critically ill patients. *American Journal of Surgery* **136**, 31–36.
20. Scher K.S., Sarap M.D., Jaggers R.L. (1986) Acute acalculous cholecystitis complicating aortic aneurysm repair. *Surgery Gynecology and Obstetrics* **163**, 475–478.

21. McDermott, M.W., Scudamore, C.H., Boileau, L.O., Snelling C.F.T. and Kramer, T.A. (1985). Acute cholecystitis: its role as a complication of major burn injury. *Canadian Journal of Surgery* **28**, 529–533.
22. Jonsson, P.E., Andersson, A. (1976). Postoperative acute acalculous cholecystitis. *Archives of Surgery* **111**, 1097–1101.
23. Glenn, F. and Becker C.G. (1982). Acalculous cholecystitis, an increasing entity. *Annals of Surgery* **195**, 131–136.
24. Howard, R.J. (1981). Acute acalculous cholecystitis. *American Journal of Surgery* **141**, 194–198.
25. Gutman, H., Kott, I., Haddad, M. and Reiss, R. (1988). Changing trends in surgery for benign gallbladder disease. *American Journal of Gastroenterolology* **83**, 545–548.
26. Thomas, C.G. and Womack, N.A. (1952). Acute cholecystitis, its pathogenesis and repair. *Archives of Surgery* **64**, 590–600.
27. Heuman, R., Norrby, S., Sjodahl, R., Tiselius, H.G., Tagesson, C. (1980). Altered gallbladder bile composition in gallstone disease. Relation to gallbladder wall permeability. *Scandinavian Journal of Gastroenterology* **15**, 581–586.
28. Womack, N.A. and Bricker, E.M. (1942). Pathogenesis of cholecystitis. *Archives of Surgery*, **44**, 658–676.
29. Gullick, H.D. (1960). A roentgenologic study of gallbladder evacuation following non-biliary tract surgery. *Annals of Surgery* **151**, 403–408.
30. Glanville, J.N. and Duthie, H.L. (1964). Contraction of the gallbladder before and after total abdominal vagotomy. *Clinical Radiology* **15**, 350–354.
31. Ihasz, M. and Griffith, C.A. 1981. Gallstones after vagotomy. *American Journal of Surgery* **141**, 48–50.
32. Ivy, A.C. (1947). Motor dysfunction of the biliary tract, an analytical and critical consideration. *American Journal of Roentgenology* **57**, 1–11.
33. Joehl, R.J., Koch, K.L. and Nahrwold D.L. (1984) Opioid drugs cause bile duct obstruction during hepato-biliary scans. *American Journal of Surgery* **147**, 134–138.
34. Flancbaum, L., Majerus, T.C. and Cox E.F. (1985). Acute posttraumatic acalculous cholecystitis. *American Journal of Surgery* **150**, 252–256.
35. Johnson, E.E. and Hedley-Whyte J. (1975). Continuous positive- pressure ventilation and choledochoduodenal flow resistance. *Journal of Applied Physiology* **39**, 937–942.
36. Roslyn, J.J., Pitt, H.A., Mann, L.L., Ament, M.E. and DenBesten, L. (1983). Gallbladder disease in patients on long-term parenteral nutrition. *Gastroenterology* **84**, 148–154.
37. Glenn, F. and Wantz, G.E. (1956). Acute cholecystitis following the surgical treatment of unrelated disease. *Surgery Gynecology and Obstetrics* **102**, 145–153.
38. Levin, M.N. (1961). Acute cholecystitis following surgery unrelated to the biliary tract. *Journal of the American Medical Association* **177**, 644–646.
39. Orlando, R., Gleason E. and Drezner, A.D. (1983). Acute acalculous cholecystitis in the critically ill patient. *American Journal of Surgery* **145**, 472–476.
40. Doty, J.E., Pitt, H.A., Porter-Fink, V. and Denbesten, L. (1985). Cholecystokinin prophylaxis of parenteral nutrition-induced gallbladder disease. *Annals of Surgery* **201**, 76–80.
41. Nobusawa, S. and Endo, M., 1988. Prophylaxis of postoperative biliary stasis: a retrospective and prospective study. *American Journal of Gastroenterology* **83**, 46–54.
42. Howard, J.M., Milford, M.T. and DeBakey, M.E. (1952). The significance of the sympathetic nervous system in acute cholecystitis. *Surgery* **32**, 251–257.
43. Golden, G.T., Sears, H.F. and Wangensteen, S.L. (1973). Post-traumatic cholecystitis. *American Surgeon* **39**, 275–278.
44. Williamson, R.C.N. (1990). Acute cholecystitis, calculous and acalculous. In Emergency Abdominal Surgery eds. Williamson RCN, Cooper MJ, Churchill Livingstone, Edinburgh, in press.
45. Ternberg, J.L. and Keating, J.P. (1975). Acute acalculous cholecystitis, complication of other diseases in childhood. *Archives of Surgery* **110**, 543–547.
46. Becker, C.G., Dubin, T. and Glenn, F (1980) Induction of acute cholecystitis by activation of factor XII. *Journal of Experimental Medicine* **151**, 81–90.
47. Kaminski, D.L., Deshpende, Y. Thomas, L. Qualy, J. and Blank, W. (1985). Effect of oral ibuprofen on formation of prostaglandins E & F by human gallbladder muscle and mucosa. *Digestive Diseases and Sciences* **30**, 933–940.
48. Kaminski, D.L., Deshpende, Y.G. and Thomas, L.A. (1987). The role of prostaglandins E and F in acalculous gallbladder disease. *Hepato-gastroenterology* **34**, 70–73.
49. Sjodahl, R. and Tagesson, C. (1983). On the development of primary acute cholecystitis. *Scandinavian Journal of Gastroenterology* **18**, 577–579.
50. Becker, C.D., Burckhardt, B. and Terrier, F. (1986). Ultrasound in postoperative acalculous cholecystitis. *Gastrointestinal Radiology* **11**, 47–50.

51. Beckman, I., Dash, N., Sefczek, R.J., Lupetin, A.R., Anderson, J.S., Diamond, D.L., and Young, J.C. (1985). Ultrasonographic findings in acute acalculous cholecystitis. *Gastrointestinal Radiology* **10**, 387–389.
52. Shlaer, W.J., Leopold, G.R. and Scheible, F.W. (1980). Sonography of the thickened gallbladder wall: a non-specific finding. *American journal of Roentgenology* **136**, 337–339.
53. Cohan, R.H., Mahony, B.S., Bowie, J.D., Cooper, C., Baker, M.E., Illescas, F.F. (1987). Striated intramural gallbladder lucencies on US studies. Predictors of acute cholecystitis. *Radiology* **164**, 31–35.
54. Siskind, B.N., Hawkins, H.B., Cinti, D.C. Zeman, R.K. and Burrell, M.I. (1987). Gallbladder perforation. An imaging analysis. *Journal of Clinical Gastroenterology* **9**, 670–678.
55. Warshauer, D., Scott, G. and Gottschalk, A. (1987). Focal acute acalculous cholecystitis. *American Journal of Roentgenology* **149**, 505–506.
56. Mirvis, S.E., Vainright, J.R., Nelson, A.W., Johnston, G.S., Shorn, R., Rodriguez, A. and Whitley, N.O. (1986). The diagnosis of acute acalculous cholecystitis: a comparison of sonography, scintigraphy and CT. *American Journal of Roentgenology* **147**, 1171–1175.
57. Fink-Bennett, D., Freitas, J.E., Ripley, S. D. and Bree, R.L. (1985). The sensitivity of hepatobiliary imaging and real-time ultrasonography in the detection of acute cholecystitis. *Archives of Surgery* **120**, 904–906.
58. Ramanna, L., Salimpour, P., Brachman, M., Tanasescu, D., Berman, D., and Waxman, A. (1982) Evaluation of Tc-99m PIPIDA scintigraphy in acalculous cholecystitis. *Journal of Nuclear Medicine* **23**, P90 (Abstract).
59. Shuman, W.P., Rogers, J.V., Rudd, T.G., Mack, L.A., Plumley, T. and Larson, E.B. (1984). Low sensitivity of sonography and cholescintigraphy in acalculous cholecystitis. *American Journal of Roentgenology* **142**, 531–534.
60. Weissman, H.S., Berkowitz, D., Fox, M.S., Gliedman, M.L., Rosenblatt, R., Sugarman, L.A. and Freeman L.M., (1983). The role of technetium-99m imidoacetic acid (IDA) cholescintigraphy in acute acalculous cholecystitis. *Radiology* **146**, 177–180.
61. Cabellon, S., Brown, J.M. and Cavanagh, D.G. (1984). Accuracy of the hepatobiliary scan in acute cholecystitis. *American Journal of Surgery* **148**, 607–608.
62. Zeman, R.K., Burrell, M.I., Cahow, C.E., and Caride, V. (1981). Diagnostic utility of cholescintigraphy and ultrasonography in acute cholecystitis. *American Journal of Surgery* **141**, 446–451.
63. Eggermont, A.M., Lameris, J.S. and Jeekel, J. (1985). Ultrasound-guided percutaneous transhepatic cholecystostomy for acute acalculous cholecystitis. *Archives of Surgery* **120**, 1354–1356.
64. Datz, F.L. (1986). Utility of indium-111-labelled leucocyte imaging in acute acalculous cholecystitis. *American Journal of Roentgenology* **147**, 813–814.
65. Felice, P.R., Trowbridge, P.E. and Ferrara, J.J. (1985). Evolving changes in the pathogenesis and treatment of the perforated gallbladder. *American Journal of Surgery* **149**, 466–473.
66. Johnson, L.B. (1987). The importance of early diagnosis of acute acalculous cholecystitis. *Surgery Gynecology and Obstetrics* **164**, 197–203.
67. Bornman, P.C., Terblanche, J. (1985). Subtotal cholecystectomy for the difficult gallbladder in portal hypertension and cholecystitis. *Surgery* **98**, 1–6.
68. Bauer, T. and Steven, K. (1988). Acute acalculous cholecystitis after radical cystectomy. *Journal of Urology* **139**, 128–129.
69. Buckley, P.M. and Hunter, J.M. (1985). Acute acalculous cholecystitis following multiple skeletal trauma. *Anesthesia* **40**, 23–26.
70. Hopkinson, G.B., Crowson, M.C. and Barnes, A.D. (1985). Perforation of the acalculous gallbladder following renal transplantation. *Transplantation Proceedings* **17**, 2014–2015.
71. Schneider, P.B. (1984). Acalculous cholecystitis: a case with variable cholescintigram. *Journal of Nuclear Medicine* **25**, 64–65.
72. Laws, P. and Elliot, R.L. (1971). Postoperative acalculous gangrenous cholecystitis. *American Surgeon.* **37**, 371–374.
73. Herlin, P., Ericsson, M., Holmin, T. and Jonsson, P.E. (1982). Acute acalculous cholecystitis following trauma. *British Journal of Surgery* **69**, 475–476.
74. Shields, M.A. (1973). Acute acalculous cholecystitis: an important complication of trauma. *Journal of the Royal College of Surgeons of Edinburgh.* **18**, 83–86.
75. Siffert, G. (1965). Alithiasic cholecystopathies and chronic non-calculous cholecystitis. In: Bockus H.L. ed. Gastroenterology III 1963 Saunders Philadelphia, London 732–745.
76. Price, W.H. (1963). Gallbladder dyspepsia. *British Medical Journal* **2**, 138–141.
77. Rhind, J.A. and Watson, L. (1968). Gallstone dyspepsia. *British Medical Journal* **1**, 32.
78. Vinnard, RT (1977). The acalculous gallbladder. *American Journal of Surgery* **133**, 153–155.

79. Lee, A.W., Proudfoot, W.H. and Griffen, W.O. (1984). Acalculous cholecystitis. *Surgery Gynecology and Obstetrics* **159**, 33–35.
80. Jones, A.G. and Blair, D.W. (1978). Non calculous cholecystitis. *Scottish Medical Journal* **23**, 27–31.
81. Mackey, W.A. (1934). Cholecystitis without stone. *British Journal of Surgery* **22**, 274–295.
82. McKibbin, J.P. and McDonald, J.R. (1945). The significance of polymorphonuclear leucocytes in gallbladders. *Surgery* **17**, 319- 327.
83. Mentzer, S.H. (1926). A clinical and pathologic study of cholecystitis and cholelithiasis. *Surgery Gynecology and Obstetrics* **196**, 782–793.
84. Glenn, F. and Mannitz, H. Jr. (1956). The acalculous gallbladder. *Annals of Surgery* **144**, 670–680.
85. Raptopoulos, V., Compton, C.C., Doherty, P., Smith, E.H., D'Orsi, C.J., Patwardhan, N.A. and Goldberg, R. (1986). Chronic acalculous gallbladder disease: multi-imaging evaluation with clinical pathologic correlation. *American Journal of Roentgenology* **147**, 721–724.
86. Mauro, M.A., McCartney, W.H. and Melmed, J.R. (1982). Hepatobiliary scanning with 99m Tc-PIPIDA in acute cholecystitis. *Radiology* **142**, 193–197.
87. Stern, W. (1979). Acalculous cholecystitis: results of cholecystectomy. *Medical Journal of Australia* **1**, 155–156.
88. Ivy, A.C. and Oldberg, E. (1939). A hormone mechanism for gallbladder contraction and evacuation. *American Journal of Physiology* **86**, 599–613.
89. Torsoli, A., Ramorino, M.L., Colagrande, C. and Demaio, G. (1961). Experiments with cholecystokinin. *Acta Radiologica* **55**, 193–206.
90. Cozzolino, H.J., Goldstein, F., Greening, R.R. and Wirts, C.W. (1963). The cystic duct syndrome. *Journal of the American Medical Association* **185**, 920–924.
91. Conte, V.P., Pinotti, H.W., Brito, T. de. and Pontes, J.F. (1971). Cholecystokinin cholecystogram in the diagnosis of the cystic duct syndrome. *American Journal of Digestive Diseases* **16**, 971–975.
92. Fink-Bennett, D. DeRidder, P., Kolozsi, W., Gordon, R. and Rapp, J. (1985). Cholecystokinin cholescintigraphic findings in the cystic duct syndrome. *Journal of Nuclear Medicine* **26**, 1123–1128.
93. McFarland, J.O. and Currin, J. (1969). Cholecystokinin and the cystic duct syndrome. *American Journal of Gastroenterology* **15**, 515–522.
94. Goldstein, F., Grunt, R. and Margulies, M. (1974). Cholecystokinin cholecystography in the differential diagnosis of acalculous gallbladder disease. *American Journal of Digestive Diseases* **19**, 834–849.
95. Newman, P., Browne, M.K and Mowat, W., (1983). A simple technique for quantitative cholecystokinin HIDA scanning. *British Journal of Radiology* **56**, 500–502.
96. Topper, T.E., Ryerson, T.W. and Nora, P.F. (1980). Quantitative gallbladder imaging following cholecystokinin. *Journal of Nuclear Medicine* **21**, 694–696.
97. Davis, G.B., Berk, R.N., Scheible, F.W., Witztum, K.F., Gilmore, I.T., Strong, R.M. and Hofmann, A.S. (1982). Cholecystokinin cholecystography, sonography and scintigraphy: detection of chronic acalculous cholecystitis. *American Journal of Roentgenology* **139**, 1117–1121.
98. Nathan, M.H., Newman, A. and Murray, D.J. (1978). Normal findings in oral and cholecystokinin cholecystography. *Journal of the American Medical Association* **240**, 2271–2272.
99. Freeman, J.B., Cohen, W.N. and Denbesten. L., (1975). Cholecystokinin cholangiography and analysis of duodenal bile in the investigation of pain in the right upper quadrant of the abdomen without gallstones. *Surgery Gynecology and Obstetrics* **140**, 371–376.
100. Griffen, W.O., Bivins, B.A., Rogers, E.L., Shearer, G.R., Liebschutz, D. and Lieber, A. (1980). Cholecystokinin cholecystography in the diagnosis of gallbladder disease. *Annals of Surgery* **191**, 636–640.
101. Backlund, V. (1967). Quoted by Hedner, P. and Lunderquist, A. (1972). *American Journal of Roentgenology 116*, 320–326.
102. Rolny, P., Arleback, A., Funch-Jensen, P., Kruse, A., Ravensbaeck, J. and Jarnerot, G. (1986). Paradoxical response of sphincter of Oddi to intravenous injection of cholecystokinin or ceruletide. Manometric findings and results of treatment of biliary dyskinesia. *Gut* **27**, 1507–1511.
103. Hogan, W., Geenan, J., Dodds, W., Toouli, J., Venu, R. and Helm, J. (1982). Paradoxical motor response to cholecystokinin (CCK-OP) in patients with suspected sphincter of Oddi dysfunction. *Gastroenterology* **82**, 1085 (Abst).
104. Hedner, P. and Lunderquist, A. (1972). Use of the C-terminal octapeptide of cholecystokinin for gallbladder evacuation in cholecystography. *American Journal of Roentgenology* **116**, 320–326.
105. Parker, J.G. and Beneventano, T.C. (1970). Acceleration of small bowel contrast study by cholecystokinin. *Gastroenterology* **58**, 679–684.

106. Dinoso, V.P. Jr., Meshkinpour, H., Lorber, S.H., Gutierrez, J.G. and Chey, W.Y. (1973). Motor response of the sigmoid colon and rectum to exogenous cholecystokinin and secretin. *Gastroenterology* **65**, 438–444.
107. Broden, B. (1958). Experiments with cholecystokinin in cholecystography. *Acta Radiologica* **49**, 25–30.
108. Edholm, P. (1960). Gallbladder evacuation in the normal male induced by cholecystokinin. *Acta Radiologica* **53**, 257-265.
109. Lilja, P., Fagan, C.J., Wiener, I. Inoue, K., Watson, L.C., Rayford, P.L. and Thompson, J.C. (1982). Infusion of pure cholecystokinin in humans. *Gastroenterology* **83**, 256–261.
110. Spellman, S.J., Shaffer, E.A. and Rosenthall, L. (1979). Gallbladder emptying in response to cholecystokinin, a cholescintigraphic study. *Gastroenterology* **77**, 115–120.
111. Foss, D.C. and Laing, R.R. (1977). Detection of gallbladder disease in patients with normal oral cholecystograms. *Digestive Diseases and Sciences* **22**, 685–689.
112. Lalyre, Y., Wilson, D.E., Kidao, J., Hall, C.H. and Capek, V. (1981). Comparison of intravenous and intramuscular sincalide (C-terminal octapeptide of cholecystokinin) on gallbladder contraction in man. *Digestive Diseases and Sciences* **26**, 214–217.
113. Sargent, E.N., Meyers, H.I. and Hebsher, J. 1976. Cholecystokinetic cholecystography; efficacy and tolerance study of sincalide. *American Journal of Roentgenology* **127**, 267–271.
114. Levant, J.A. and Sturdevant, R.A.L. (1974). Use of C-terminal octapeptide of cholecystokinin in cholecystography. *American Journal of Roentgenology* **121**, 380–383.
115. Arnold, J.D., Boyer, S. and Schemano, I. (1980). Comparative cholecystokinetic effects of intramuscular ceruletide, intravenous sincalide, oral fatty meal and intramuscular placebo. *Clinical Pharmacology and Therapeutics* **27**, 245 (Abstract).
116. Davidsen, D., Jorgensen, J. (1981). Gallbladder emptying with ceruletide in oral cholecystography. *Acta Radiologica Diagnosis* **22**, 165–169.
117. Rosenquist, C.J. and Barcia, T.C. (1983). Studies of gallbladder contraction using intramuscular sincalide. *Radiology* **146**, 21–23.
118. Hederstrom, E., Forsberg, L., Herlin, P. and Holmin, T. (1988). Fatty meal provocation monitored by ultrasonography. *Acta Radiologica* **29**, 207–210.
119. Legmann, P. Guichard, J.P., Boudinet, C. and Levesque, M. (1986). Gallbladder sonography with use of a fatty meal: a study of 115 patients. *Radiology* **32**, 161P (Abstract).
120. Hopman, W.P.M., Rosenbusch, G., Jansen, J.B.M.J., de Jong, A.J.L. and Lamers, C.B.H.W., (1985). Gallbladder contraction: effects of fatty meals and cholecystokinin. *Radiology* **157**, 37–39.
121. Park, C.Y., Pae, Y.S. and Hong, S.S. (1970). Radiological studies on emptying of human gallbladder. *Annals of Surgery* **171**, 294–299.
122. Feigelson, H.H., Berk, J.D., Joyrich, M.H., Gagliardi, R.A. and Shufro, A.S. (1960). The effectiveness of oral cholecystagogues and intravenous cholecystokinin in producing bile duct visualisation during oral cholecystography. *Radiology* **75**, 268–271.
123. Dunn, F.H., Christensen, E.C., Reynolds, J., Jones, V. and Fordtran, J.S. (1974). Cholecystokinin cholecystography. *Journal of the American Medical Association* **228**, 997–1003.
124. Rajagopolan, E. and Pickleman, J. (1982).Biliary colic and functional gallbladder disease. *Archives of Surgery* **117**, 1005-1008.
125. Byrne, P., Hunter, G.J.S. and Vallon, A. (1985). Cholecystokinin cholecystography: a three-year prospective trial. *Clinical Radiology* **36**, 499–502.
126. Reid, DRK, Rogers, I.M. and Calder, J.F. (1975). The cholecystokinin test: an assessment. *British Journal of Surgery* **62**, 317–319.
127. Valberg, L.S., Jabbari, M., Kerr, J.W., Curtis, A.C., Ramchand, S. and Prentice, R.S.A. (1971). Biliary pain in young women in the absence of gallstones. *Gastroenterology* **60**, 1020–1026.
128. Harvey, R.F. and Read, A.E. (1973). Effect of cholecystokinin on colonic motility and symptoms in patients with the irritable bowel syndrome. *Lancet* **1**, 1–3.
129. Neschis, M., King, M.C. and Murphy, R.A.(1978). Cholecystokinin cholecystography in the diagnosis of acalculous extrahepatic biliary tract disorders. *American Journal of Gastroenterology* **70**, 593–599.
130. Nora, P.F., Davis, R.P. and Fernandez, M.J. (1984). Chronic acalculous gallbladder disease: a clinical enigma. *World Journal of Surgery* **8**, 106–112.
131. Burnstein, M.J., Vassal, K.P. and Strasberg, S.M. (1982). Results of combined biliary drainage and cholecystokinin cholecystography in 81 patients with normal oral cholecystograms. *Annals of Surgery* **196**, 627–632.
132. Einarsson, K., Angelin, B., Kelter, U., Nyberg, B. and Sonnenfeld, T. (1986). Biliary colic without evidence of gallstones: diagnosis, biliary lipid metabolism and treatment. *Acta Chirurgica Scandinavica*. **Suppl. 530**, 31–34.

133. Goldberg, H.I. (1976). Cholecystokinin cholecystography. *Seminars in Roentgenology*, **11**, 175–179.
134. Moskovitz, M., Min, T.C., Gavaler, J.S. (1986). The microscopic examination of bile in patients with biliary pain and negative imaging tests. *American Journal of Gastroenterology* **81**, 329–333.
135. Nathan, M.H., Newman, A., Murray, D.J. and Camponovo, R. (1970). Cholecystokinin cholecystography. *American Journal of Roentgenology* **110**, 240–251.
136. Nora, P.F., McCarthy, W. and Sanez, N. (1974). Cholecystokinin cholecystography in acalculous gallbladder disease. *Archives of Surgery* **108**, 507–511.
137. Rhodes, M., Lennard, T.W.J., Farndon, J.R. and Taylor, R.M.R. (1988). Cholecystokinin (CCK) provocation test: long-term follow-up after cholecystectomy. *British Journal of Surgery*. **75**, 951–953.
138. Sunderland, G.T. and Carter, D.C. (1987). Cholecystokinin provocation does not predict outcome in acalculous biliary pain. *British Journal of Surgery* **74**, 1157–1158.
139. Sykes, D. (1982). The use of cholecystokinin in diagnosing biliary pain. *Annals of the Royal College of Surgeons* **64**, 114-116.
140. Madsen, P.E.R., Andersen, J.F., Jensen, K.B. *et al* (1981). Quoted by Thornell E. (1985). *British Journal of Surgery* **72**, 585.
141. Thornell, E. (1985). Cholecystokinin (CCK) cholecystography and biliary pain. *British Journal of Surgery* **72**, 585.
142. Windsor, C.W.O. and Forrest, J. (1982). Cholecystokinin in diagnosis of biliary pain. *Annals of the Royal College of Surgeons* **64**, 280.
143. Sunderland, G.T. and Carter, D.C. (1988). Clinical application of the cholecystokinin provocation test. *British Journal of Surgery* **75**, 444–449.
144. Berk, R.N. (1977). Cholecystokinin cholecystography in the diagnosis of chronic acalculous cholecystitis and biliary dyskinesia. *Gastrointestinal Radiology* **1**, 325–330.
145. Brugge, W.R., Brand, D.L., Atkins, H.L., Lane, B.P. and Abel, W.G., (1986). Gallbladder dyskinesia in chronic acalculous cholecystitis. *Digestive Diseases and Sciences* **31**, 461–467.
146. Khuroo, M.S. and Zargar, S.A. (1985). Biliary ascariasis. A common cause of biliary and pancreatic disease in an endemic area. *Gastroenterology* **88**, 418–423.
147. Cohen, E.K., Stringer, D.A., Smith, C.R. and Daneman, A. (1986). Hydrops of the gallbladder in typhoid fever as demonstrated by sonography. *Journal of Clinical Ultrasound* **14**, 633–635.
148. Glenn, F. and Hill, M.R. Jr. (1954). Primary gallbladder disease in children. *Annals of Surgery* **139**, 302–311.
149. Thomas, W.E.G, Thornton, J.R. and Thompson, M.H. (1981). Staphylococcal acalculous cholecystitis. *British Journal of Surgery* **68**, 136.
150. McKiernan, J., O'Brien, D.J. and Dundon, S. (1976). Leptospirosis and acalculous cholecystitis. *Journal of the Irish Medical Association* **69**, 71–72.
151. Rappaport, I., Albukerk, J. and Schneider, I.J. (1975), Schistosomal cholecystitis. *Archives of Pathology* **99**, 227–228.
152. Andersson, A., Bergdahl, L. and Boquist, L. (1971). Acalculous cholecystitis. *American Journal of Surgery* **122**, 3–7.
153. Friberg, J., Sonstabo, R., Joe, G.T., Goes, E. and Osteaux, M. (1987). Acalculous cholecystitis as a complication of hepatitis. *European Journal of Radiology* **7**, 153.
154. May, R.E., Strong, R. (1971). Acute emphysematous cholecystitis. *British Journal of Surgery* **58**, 453–458.
155. Rosoff, L. Meyers, H. (1966). Acute emphysematous cholecystitis. An analysis of ten cases. *American Journal of Surgery* **111**, 410–423.
156. Kavin, H., Jonas, R.B., Chowdhury, L. and Kabins, S. (1986). Acalculous cholecystitis and cytomegalovirus infection in the acquired immunodeficiency syndrome. *Annals of Internal Medicine* **104**, 53–54.
157. Robinson, G., Wilson, S.E. and Williams, R.A. (1987). Surgery in patients with acquired immunodeficiency syndrome. *Archives of Surgery* **122**, 170–175.
158. Aaron, J.S., Wynter, C.D., Kirton, O.C. and Simko, V. (1988). Cytomegalovirus associated with acalculous cholecystitis in a patient with acquired immune deficiency syndrome. *American Journal of Gastroenterology* **83**, 879–881.
159. Radin, D.R., Cohen, H. and Halls, J.M. (1987). Acalculous inflammatory disease of the biliary tree in acquired immunodeficiency syndrome: CT demonstration. *Journal of Computer Assisted Tomography* **11**, 775–778.
160. Dolmatch, B.L., Laing, F.C., Federle, M.P., Jeffry, R,B. and Cello, J. (1987). AIDS-related cholangitis; radiographic findings in nine patients. *Radiology* **163**, 313–316.
161. Thorbjarnarson, B. (1960). Carcinoma of the gallbladder and acute cholecystitis. *Annals of Surgery* **151**, 241–244.

162. Hoerr, S.O. and Hazard, J.B. (1966). Acute cholecystitis without gallbladder stones. *American Journal of Surgery* **111**, 47–55.
163. Andry, G., Turnbull, A.D., Botet, J. and Kurtz, R.C. (1986). Cholesonographic characteristics of cystic duct metastasis causing acute aealculous cholecystitis: case report. *Journal of Surgical Oncology* **31**, 178–181.
164. Ostick, D.G. and Haqqani, M.T. (1976). Obstructive cholecystitis due to metastatic melanoma. *Postgraduate Medical Journal* **52**, 710–712.
165. Dainko, E.A. (1970). Acalculous cholecystitis due to Hodgkin's disease. *California Medicine* **112**, 28–30.
166. Ashby, B.S. (1965). Acute and recurrent torsion of the gallbladder. *British Journal of Surgery* **52**, 182–184.
167. Greenwood, R.K. (1963). Torsion of the gallbladder. *Gut* **4**, 27–29.
168. Schlinkert, R.T., Mucha, P., Farnell, M.B. (1984). Torsion of the gallbladder. *Mayo Clinic Proceedings* **59**, 490–492.
169. Recht, W (1952). Torsion of a double gallbladder. A report of a case and a review of the literature. *British Journal of Surgery* **39**, 342–344.
170. Goldman, G., Rafael, A.J. and Hanoch, K. (1985). Acute acalculous cholecystitis due to an incarcerated epigastric hernia. *Postgraduate Medical Journal* **61**, 1017–1018.
171. Quinn, S.F., Fazzio, F. and Jones, E. (1987). Torsion of the gallbladder: findings on CT sonography and the role of percutaneous cholecystostomy. *American Journal of Roentgenology* **148**, 881–882.
172. Livolsi, V.A., Perzin K.H. and Porter M. (1973). Polyarteritis nodosa of the gallbladder presenting as acute cholecystitis. *Gastroenterology* **65**, 115–123.
173. Swanepoel, C.R., Floyd, A., Allison, H., Learmonth, G.M., Cassidy M.J.D. and Pascoe M.D. (1983). Acute acalculous cholecystitis complicating systemic lupus erythematosus: case report and review. *British Medical Journal* **286**, 251–252.
174. Slovis, T.L., Hight, D.W., Phillippart, A.I. and Dubois, R.S. (1980). Sonography in the diagnosis and management of hydrops of the gallbladder in children with mucocutaneous lumph node syndrome. *Pediatrics* **65**, 789–794.
175. Tanaka, K., Shimada, M., Hattori, M., Utsunomiya, T. and Oya, N. (1985). Sjögren's syndrome with abnormal manifestations of the gallbladder and central nervous system. *Journal of Pediatric Gastroenterology and Nutrition* **4**, 148–151.
176. Thorpe, M.E.C., Scheuer, P.J. and Sherlock, S. (1967). Primary sclerosing cholangitis, the biliary tree and ulcerative colitis. *Gut* **8**, 435–438.
177. Copeman, P.W.M. and Medd, W.E. (1967). Diffuse systemic sclerosis with abnormal liver and gallbladder. *British Medical Journal* **3**, 353–354.
178. Lafon, P.C., Reed, K. and Rosenthal, D. (1985). Acute cholecystitis associated with hepatic arterial infusion of floxuridine. *American Journal of Surgery* **150**, 687–689.
179. Carrasco, C.H., Freeny, P.C., Chuang, V.P. and Wallace, S. (1983). Chemical cholecystitis associated with hepatic artery infusion chemotherapy. *American Journal of Roentgenology* **141**, 703–706.
180. Pietrafitta, J.J., Anderson, B.G., O'Brien, M.J. and Deckers, P.J. (1986). Cholecystitis secondary to infusion chemotherapy. *Journal of Surgical Oncology* **31**, 287–293.
181. Hohn, D.C., Rayner, A.A., Economou, J.S., Ignoffo, R.J., Lewis, B.J. and Stagg, R.J., (1986). Toxicities and complications of implanted pump hepatic arterial and intravenous floxuridine infusion. *Cancer* **57**, 465–470.
182. Schaad, U.B., Wedgewood-Krucko, J. and Tschaeppeler, H. (1988). Reversible ceftriaxone-associated biliary pseudolithiasis in children. *Lancet* **2**, 1411–1413.
183. Pieretti, R., Auldist, A.W. and Stephens, C.A. (1975). Acute cholecystitis in children. *Surgery Gynecology and Obstetrics* **140**, 16–18.
184. Ulin, A.W., Nosal, J.L. and Martin, W.L. (1952). Cholecystitis in childhood: associated obstructive jaundice. *Surgery* **31**, 312–326.
185. Sneider, S.E. and Winslow, O.P. (1962). Cholecystitis and cholelithiasis associated with pancreatitis in a child. *Journal of the American Medical Association* **182**, 302–303.
186. Andrassy, R.J., Treadwell, T.A., Ratner, I.A. and Buckley, C.J. Gallbladder disease in children and adolescents. *American Journal of Surgery* **132**, 19–21.
187. Bloom, R.A. and Swain, V.A.J. (1966). Non-calculous distension of the gallbladder in childhood. *Archives of Diseases in Childhood.* **41**, 503–508.
188. Bowen, A., (1984). Acute gallbladder dilatation in a neonate: emphasis on ultrasonography. *Journal of Pediatric Gastroenterology and Nutrition* **3**, 304–308.

189. Thurston, W.A., Kelly, E.N. and Silver, M.M. (1986). Acute acalculous cholecystitis in a premature infant treated with parenteral nutrition. *Canadian Medical Association Journal* **135**, 332–334.
190. Rumley, T.O. and Rodgers, B.M. (1983). Hydrops of the gallbladder in children. *Journal of Pediatric Surgery* **18**, 138–140.
191. Neu, J., Arvin, A. and Ariagno, R.L. (1980). Hydrops of the gallbladder. *American Journal of Diseases of Children* **134**, 891--893.
192. Traynelis, V.C. and Hrabovsky, E.E. (1985). Acalculous cholecystitis in the neonate. *American Journal of Diseases of Children* **139**, 893–895.

*(Accepted by S. Bengmark 29 May 1989)*

